# Bacteriophage therapy reduces *Staphylococcus aureus* in a porcine and human *ex vivo* burn wound infection model

**DOI:** 10.1128/aac.00650-24

**Published:** 2024-08-13

**Authors:** Michèle M. Molendijk, Bouke K. H. L. Boekema, Kirby R. Lattwein, Marcel Vlig, Lonneke G. M. Bode, Marion P. G. Koopmans, Annelies Verbon, Miranda de Graaf, Willem J. B. van Wamel

**Affiliations:** 1 Department Medical Microbiology and Infectious Diseases, Erasmus MC, Rotterdam, the Netherlands; 2 Department of Viroscience, Erasmus MC, Rotterdam, the Netherlands; 3 Association of Dutch Burn Centres, Beverwijk, the Netherlands; 4 Department of Cardiology, Erasmus MC, Rotterdam, the Netherlands; 5 Department of Internal Medicine, UMC Utrecht, Utrecht, the Netherlands; The Peter Doherty Institute for Infection and Immunity, Melbourne, Australia

**Keywords:** burn wound infection, *Staphylococcus aureus*, bacteriophage therapy, *ex vivo *models, human, porcine

## Abstract

Burn wounds are a major burden, with high mortality rates due to infections. *Staphylococcus aureus* is a major causative agent of burn wound infections, which can be difficult to treat because of antibiotic resistance and biofilm formation. An alternative to antibiotics is the use of bacteriophages, viruses that infect and kill bacteria. We investigated the efficacy of bacteriophage therapy for burn wound infections, in both a porcine and a newly developed human *ex vivo* skin model. In both models, the efficacy of a reference antibiotic treatment (fusidic acid) and bacteriophage treatment was determined for a single treatment, successive treatment, and prophylaxis. Both models showed a reduction in bacterial load after a single bacteriophage treatment. Increasing the frequency of bacteriophage treatments increased bacteriophage efficacy in the human *ex vivo* skin model, but not in the porcine model. In both models, prophylaxis with bacteriophages increased treatment efficacy. In all cases, bacteriophage treatment outperformed fusidic acid treatment. Both models allowed investigation of bacteriophage-bacteria dynamics in burn wounds. Overall, bacteriophage treatment outperformed antibiotic control underlining the potential of bacteriophage therapy for the treatment of burn wound infections, especially when used prophylactically.

## INTRODUCTION

Annually, an estimated 11 million people require medical treatment for burn wounds globally. Mortality rates of burn wounds requiring treatment are high and can even reach up to 25%, depending on many factors, including age, socioeconomic factors, and the size of the wound (1). It is estimated that bacterial infections account for 75% of this mortality (2, 3). Burn wounds are especially prone to infection due to the disruption of the skin barrier, leading to impaired host defenses. Consequently, wound healing is delayed, and hospital stays are prolonged (4). *Staphylococcus aureus* (*S. aureus*) is one of the major causative agents of burn wound infections. To treat burn wound infections, wound debridement is typically performed to remove the affected tissue, and topical antimicrobial therapy is initiated, usually together with systemic antibiotic administration (5). For topical treatment, antimicrobial ointments (e.g., silver sulfadiazine) or antibiotics are often used, with mupirocin and fusidic acid specifically effective against staphylococcal infections (5, 6). However, the high prevalence of antimicrobial resistance and the tendency of *S. aureus* to form biofilms complicate burn wound infection treatment with antibiotics (4).

A potential alternative to antibiotics is the use of bacteriophages (phages), which are viruses that can infect and kill bacteria (7). Although phage therapy is commonly practiced in Eastern Europe, its efficacy remains to be determined due to the lack of well-organized clinical trials (8). Thus far, only one double-blind phase 1/2 clinical trial on phage therapy for burn wound infections has been published. In this trial, safety and efficacy of phage therapy for burn wound infections of *Pseudomonas aeruginosa* were investigated. However, the trial ended early due to the insufficient efficacy of the phage treatment. The high bacterial loads in the phage-treated group were attributed to the low phage titers used for treatment, resulting from a steep decrease in phage titer after manufacturing (9). Phage therapy for burn wound infections caused by *S. aureus* has only been described in one small clinical trial, which included nine participants (10). This trial showed no differences in bacterial load between the burn wound, half treated with phages and the other half treated with the standard of care. However, the authors indicated that this might be due to practical issues with the route of administration of the phages, the late inclusion of the patients, and prior treatment with antibiotics before inclusion in the clinical trial.

Although phage therapy was not found to be effective in these two human trials, it has been shown to be effective in multiple animal models. For example, phage treatment of *Klebsiella pneumoniae* burn wound infections successfully reduced bacterial load and increased survival in BALB/c mice (11, 12). In addition, phage therapy accelerated wound healing of burn wounds infected with *Acinetobacter baumannii* in rats (13). Animal studies on *S. aureus* burn wound infections have not been published to date. Despite the promising results of phage efficacy testing in animal models, they do come with many limitations such as high costs and various ethical concerns. *Ex vivo* models lack most of these limitations while still representing the complexity of the *in vivo* microenvironment, something that often is lacking for *in vitro* assays (14).

In this study, two *ex vivo* burn wound models were used to investigate phage therapy efficacy against *S. aureus* burn wound infections. First, a human *ex vivo* skin model, first described by Boekema et al. (15), was adapted for this purpose (15, 16). Second, an *ex vivo* model using porcine skin was used, which is a well-established representative of human skin due to its structural similarities (14, 17). Phage treatment efficacy of single and successive treatments and prophylaxis were assessed in both models, thereby providing insight into the most effective utilization of phages for therapy. Moreover, differences between the human and porcine *ex vivo* models were investigated.

## RESULTS

### Phage efficacy over time after a single phage application

The efficacy of phages in treating *S. aureus* burn wound infections was investigated in both the porcine and human *ex vivo* models. For this purpose, two bacteriophages, phage ISP and RPCSa2, were selected. Both phages showed high *in vitro* efficacy against methicillin-resistant *S. aureus* LUH14616, as determined by an optical density assay (Fig. S1A). Three phage concentrations were used for treatment: 10^6^, 10^7^, and 10^8^ pfu/mL, corresponding to titers that have been used in animal models and human trials (9–13). In addition to phage treatment, antibiotic treatment using fusidic acid was included. Fusidic acid was applied at 30 times its minimal inhibitory concentration (MIC) (Fig. S1B). Phage-antibiotic synergy between both phages and fusidic acid was assessed using the method of Gu Liu et al. (18); however, no phage-antibiotic synergy was observed (data not shown).

**Fig 1:**
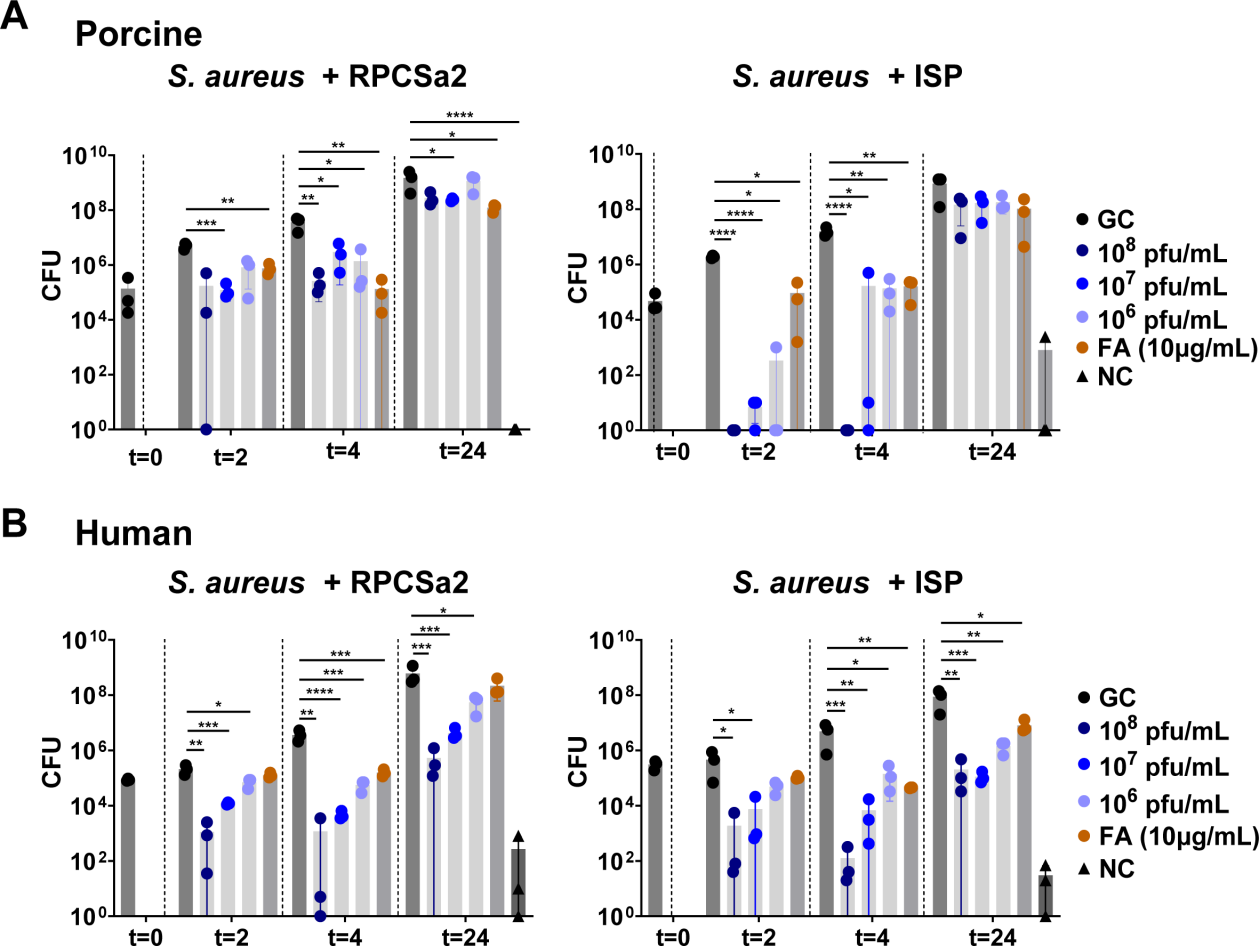
Single phage treatment of burn wound infections. *S. aureus* LUH14616 was added to burn wounds on either (**A**) porcine skin or (**B**) human skin. The burn wound infection was treated once with either phage ISP or RPCSa2 at different concentrations, fusidic acid (FA), or PBS (growth control, GC). As a negative control (NC), burned skin without the addition of bacteria was included. Each condition was tested in triplicate. Colony forming units (CFU) were determined 2, 4, and 24 h after treatment. The bacterial load of each treated sample was compared with the GC using a *t*-test, and significant decreases in CFU are indicated with (*P ≤ 0.05; **P ≤ 0.01; ***P ≤ 0.001; ****P ≤ 0.0001).

To gain insights into phage efficacy over time, colony forming units (CFU) were determined at 2, 4, and 24 h post-treatment (Fig. 1). In both models, bacterial loads in the untreated growth control gradually increased over time, reaching approximately 10^8^ and 10^9^ CFU per piece of human and porcine skin, respectively. No or very low bacterial loads (10^0^ to 10^2^ CFU) were present on the negative control skins to which no bacteria or treatment was added. Overall, similar patterns in phage efficacy were seen for the porcine and human *ex vivo* models (Fig. 1). A dose-dependent effect was observed for both phages, with the highest efficacy measured at 2 and 4 h after treatment. Despite the concentration of fusidic acid being over 30 times its MIC, it did not outperform the phage treatments but had a similar (on porcine skin) or reduced (on human skin) efficacy compared with the lowest phage concentration. Differences between the two models were also observed. There was no effect of the phage treatment after 24 h in the porcine model, but there was a significant reduction of bacterial loads in the human skin model. In addition, clear differences in phage efficacy between phage ISP and RPCSa2 were observed in the porcine model. At 2 and 4 h after treatment, phage ISP reduced the bacterial loads to the detection limit, whereas RPCSa2 treatment only resulted in a reduction to 10^6^ CFU (Fig. 1A). In the human skin model, this discrepancy between the two phages was less pronounced (Fig. 1B).

### Treatment efficacy of successive phage application

**Fig 2:**
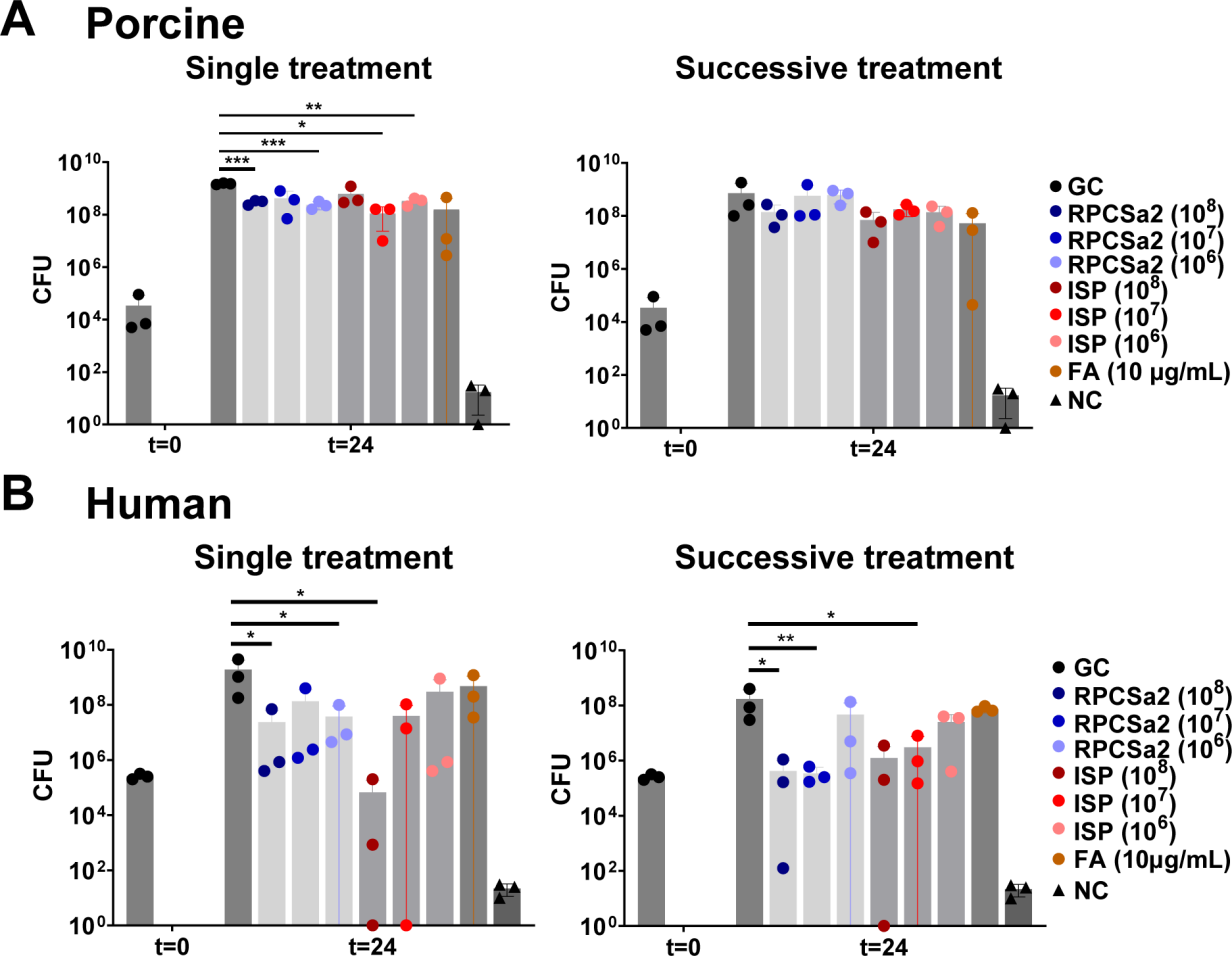
Successive phage treatment of burn wound infections. *S. aureus* LUH14616 was added to burn wounds on either (**A**) porcine skin or (**B**) human skin, which were treated with phage ISP or RCPSa2 at different concentrations of fusidic acid (FA). For successive treatment, the burn wounds were treated successively at 1, 4, and 7 h after the application of *S. aureus*. An untreated control as growth control (GC) and burned skin without the addition of bacteria as negative control (NC) were included. Each condition was tested in triplicate. CFU were determined after 24 h. Bacterial load of each treated sample was compared to the GC using a *t*-test, and significant decreases in CFU are indicated with (*P ≤ 0.05; **P ≤ 0.01; ***P ≤ 0.001; ****P ≤ 0.0001).

Next, the efficacy of successive phage applications was investigated. Since the largest reduction of *S. aureus* was observed at 2 and 4 h post-treatment (Fig. 1), in this experiment, the burn wounds were treated, every 3 h (three times in total) with either phages or fusidic acid, and CFU were determined after 24 h (Fig. 2).

For both models, the treatment efficacy of fusidic acid was equivalent to that of the lowest phage concentrations. For the porcine model, successive applications of phage ISP, RPCSa2, or fusidic acid did not result in an increase in treatment efficacy compared with a single application (Fig. 2A). Similar to a single phage application, the treatment efficacy of successive application of both the phages and fusidic acid was higher for human skin compared with porcine skin (Fig. 2B). Although successive applications on the human skin did not further improve the treatment efficacy of ISP and fusidic acid, successive applications of RPCSa2 (10^7^ pfu/mL) did result in an increased reduction of the bacterial load.

### Treatment efficacy of prophylactic phage application

**Fig 3:**
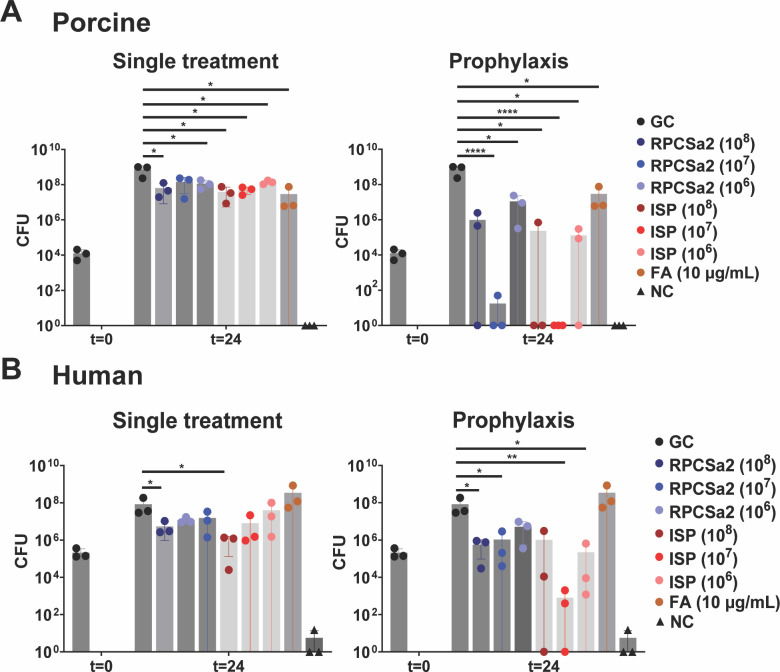
Prophylactic phage treatment of burn wound infections. *S. aureus* LUH14616 was added to burn wounds on either (**A**) porcine skin or (**B**) human skin, which were treated once with phage ISP or RCPSa2 at different concentrations of fusidic acid (FA). For prophylactic treatment, the skin was incubated with either phage ISP or RPCSa2 at different concentrations for 1 h, after which *S. aureus* was added to the burn wound. An untreated control as growth control (GC) and burned skin without the addition of bacteria as negative control (NC) were included. Each condition was tested in triplicate. CFU were determined after 24 h. Bacterial load of each treated sample was compared with the GC using a *t*-test, and significant decreases in CFU are indicated with (*P ≤ 0.05; **P ≤ 0.01; ***P ≤ 0.001; ****P ≤ 0.0001).

Finally, the potential of phage therapy as prophylaxis was evaluated (Fig. 3). For this purpose, phage ISP and RPCSa2 were applied to the skin 1 h before the addition of *S. aureus*. Twenty-four hours post-prophylactic treatment, CFU counts were compared with those of phage treatment after *S. aureus* infection. Significantly lower bacterial loads were observed with prophylactic treatment, particularly for phage ISP at 10^7^ pfu/mL. Prophylaxis on porcine skin reduced bacterial loads to 10^0^ and 10^1^ CFU for phage ISP and RPCSa2, respectively (Fig. 3A). The reduction was less pronounced on human skin, with remaining bacterial loads of 10^3^ and 10^6^ CFU (Fig. 3B).

### Visualization of phage efficacy on burn wound infections

**Fig 4:**
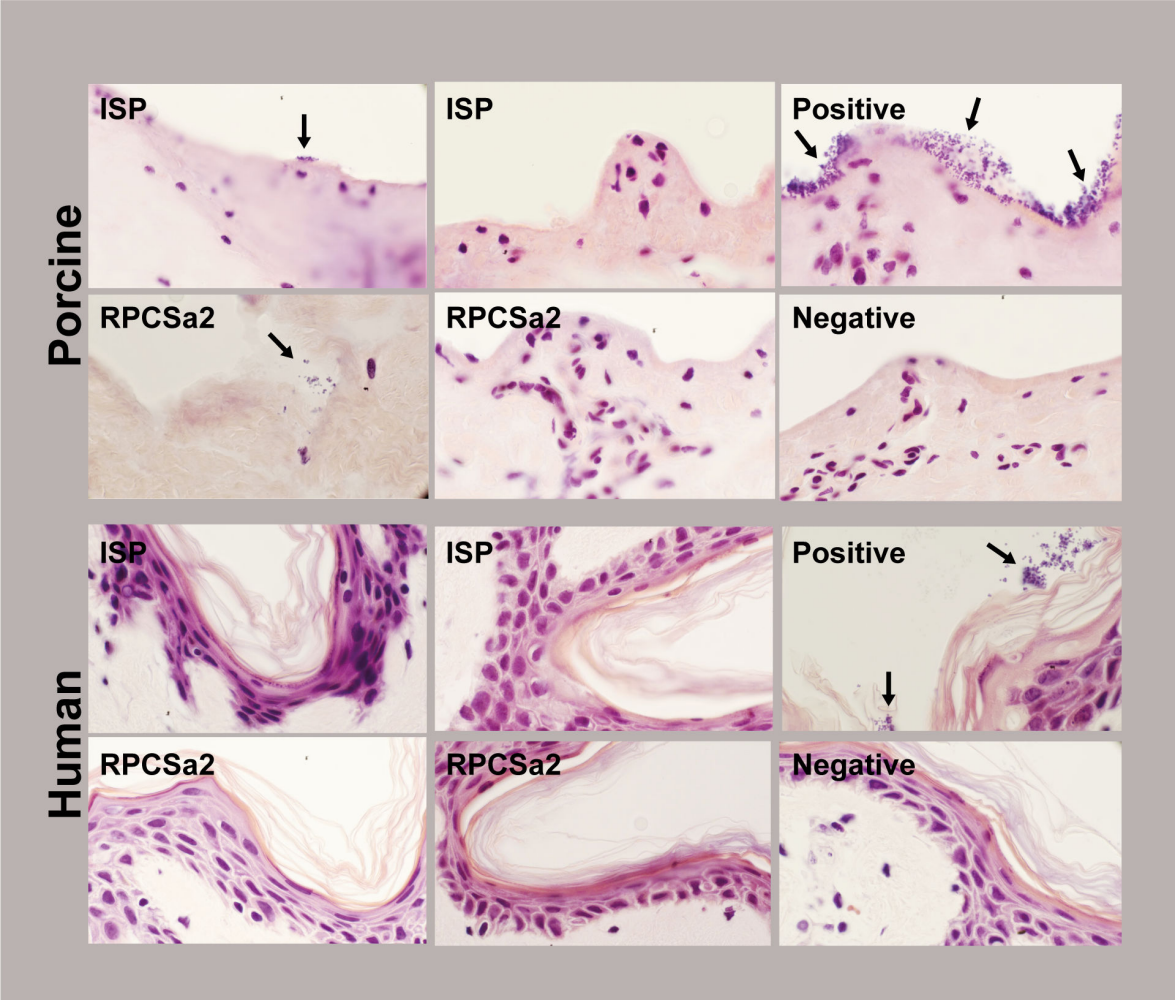
Gram-staining of porcine and human skin after phage treatment or prophylaxis. Images of Gram-stained skin tissue 24 h after phage treatment or prophylaxis with 10^8^ pfu/mL of phage ISP or RPCSa2. A positive growth control (untreated) and negative control (no bacteria added) were included for comparison. Bacteria are indicated with black arrows. Images were made at 1000× magnification.

In addition to the quantification of bacterial load, the effect of phage treatment was visualized by histochemistry (Fig. 4). Burn wounds were treated with 10^8^ pfu/mL of phage ISP or RPCSa2, either after the establishment of the *S. aureus* infection or as prophylaxis. The skin was incubated for 24 h, after which the samples were fixed in paraformaldehyde. Vertical slices were made through the burn wounds of the formalin-fixed paraffin-embedded (FFPE) skin samples, and a Gram-staining was performed. For the porcine skin model, this staining showed a dense bacterial population in the growth control, with fewer bacteria observed in the phage-treated skin and a complete absence of bacteria in the skin where phages were given as prophylaxis (Fig. 4; Table 1; Fig. S2).

**TABLE 1:** Scoring of bacteria per frame (%) of Gram-stained human or porcine burn wound infections, which received either phage treatment (T) or prophylaxis (P)

Condition^*a*^	No bacteria		1–10 bacteria		10–100 bacteria		>100 bacteria	
	Porcine	Human	Porcine	Human	Porcine	Human	Porcine	Human
GC	0	66	0	15	60	15	40	4
T-ISP	65	100	24	0	11	0	0	0
T-RPCSa2	69	100	26	0	5	0	0	0
P-ISP	100	100	0	0	0	0	0	0
P-RPCSa2	100	100	0	0	0	0	0	0
NC	100	100	0	0	0	0	0	0

^*a*^ Bacteria in each frame were scored and categorized in the following categories: no bacteria, 1 to 10 bacteria, 10 to 100, over 100 bacteria.

For the human skin model, fewer bacteria were observed in the growth control compared with the porcine skin model (Fig. 4; Table 1; Fig. S2), which correlated to the CFU data. On human skin, both the prophylactic and post-infection phage treatment resulted in an absence of bacteria. Although similar treatment efficacy patterns were observed for histochemistry and CFU data, the complete absence of bacteria was only observed for the Gram-stained tissues. It should be noted that for histochemistry, only a part of the burn wound was evaluated, whereas the CFU data included the complete burn wound and the surrounding area.

**Fig 5:**
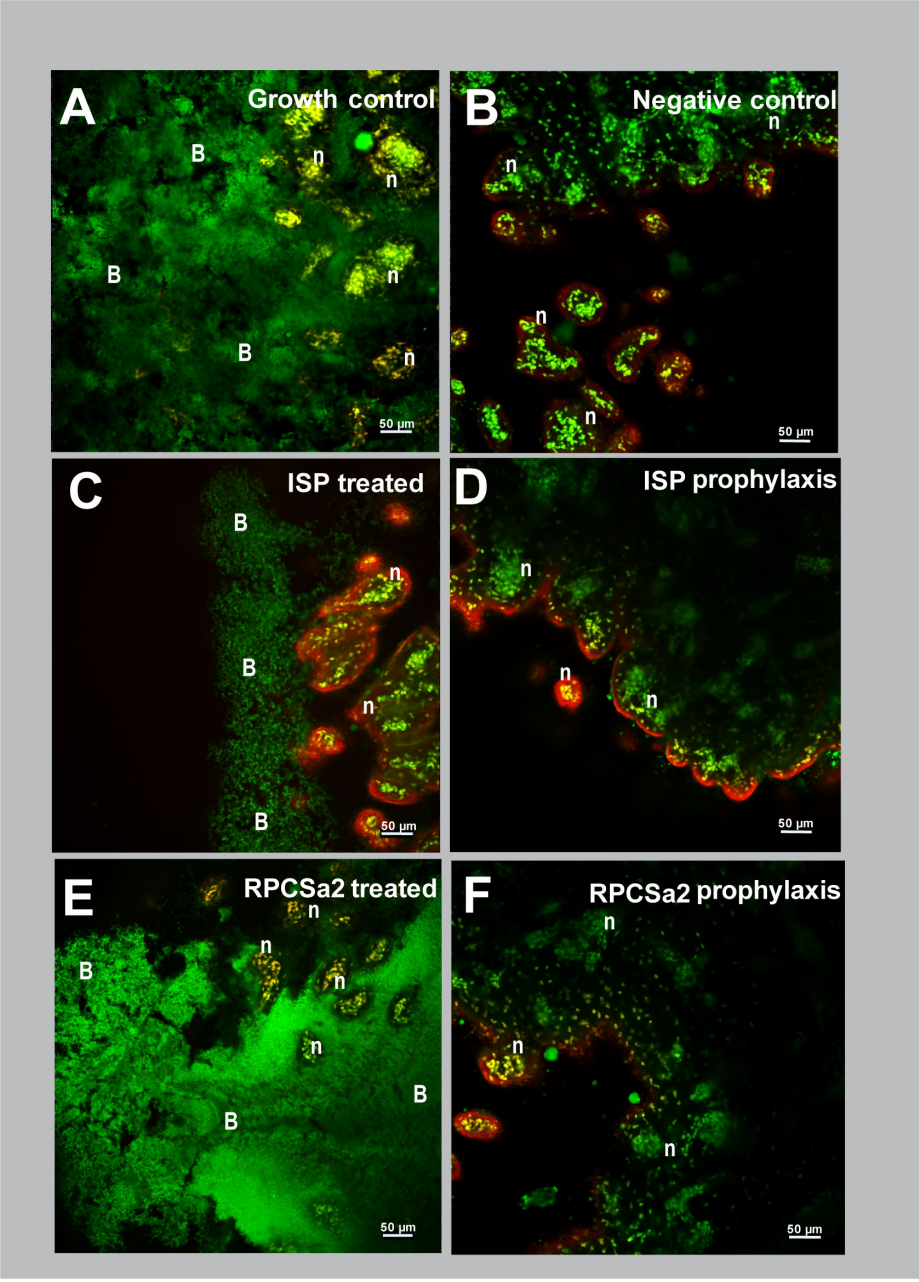
Two-dimensional confocal microscopy images of porcine skin 24 h after phage treatment or prophylaxis Images were made of porcine skin tissue 24 h post-treatment or prophylaxis. Skin was incubated with *S. aureus* and was (**A**) left untreated as growth control, (**C**) treated or (**D**) prophylactically treated with 10^8^ pfu/mL ISP, or (**E**) treated or (**F**) prophylactically treated with 10^8^ pfu/mL RPCSa2. Skin without bacteria was included as (**B**) negative control. Dead bacterial and eukaryotic cells were stained with propidium iodide (red). Living bacteria (~1 µm) or eukaryotic nuclei (~10 µm) were stained with acridine orange (green). Additionally, WGA-594 (red) stained N-acetyl glucosamine, a sugar often produced in biofilms. All images were made at 20× magnification. B, bacteria; n, eukaryotic nuclei.

**Fig 6:**
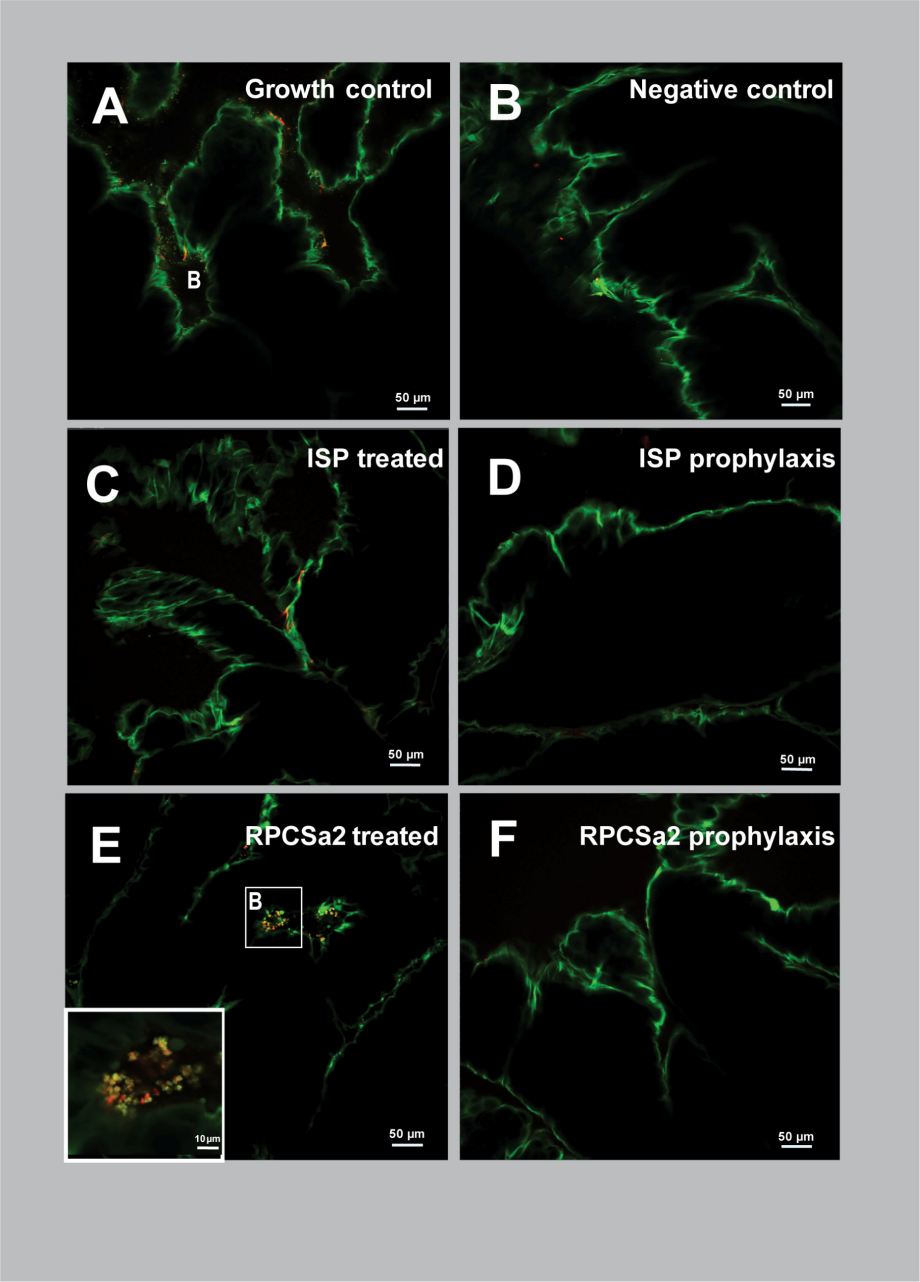
Two-dimensional confocal microscopy images of human skin 24 h after phage treatment or prophylaxis. Images were made of human skin tissue 24 h post-treatment or prophylaxis. Skin was either incubated with *S. aureus* and was (**A**) left untreated as growth control, (**C**) treated or (**D**) prophylactically treated with 10^8^ pfu/mL ISP, or (**E**) treated or (**F**) prophylactically treated with 10^8^ pfu/mL RPCSa2. Skin without bacteria was included as (B) negative control. Dead bacterial and eukaryotic cells were stained with propidium iodide (red). Living bacteria (~1 µm) or eukaryotic nuclei (~10 µm) were stained with acridine orange (green). Additionally, WGA-594 (red) stained N-acetyl glucosamine, a sugar often produced in biofilms. All images were made at 20× magnification. B, bacteria.

To investigate phage efficacy in more detail, confocal microscopy was used (Fig. 5 and 6). Following treatment and incubation, skin samples were stained to visualize living bacterial cells (~1 µm) and eukaryotic nuclei (~10 µm) (green) (19, 20), dead eukaryotic and prokaryotic cells (red), and β-N-acetyl glucosamine, which is a polymer that can be produced by biofilms (red). The two-dimensional (2D) and three-dimensional (3D) confocal images showed clear differences in skin structure between the porcine (Fig. 5B; Fig. S3B) and human (Fig. 6B; Fig. S4B) models. In the porcine skin, eukaryotic nuclei were stained green (10–20 µm), whereas these nuclei are not present in the upper layer of the human epidermis (21). Moreover, the outer layer of the porcine skin showed a clear red signal, which did not represent the circular shapes of bacterial or eukaryotic cells. Therefore, this signal was most likely caused by the natural presence of N-acetyl glucosamine on the porcine skin (22). Finally, the human skin showed high levels of unspecific binding of acridine orange (green), which was absent on the porcine skin.

For the porcine skin model, both the 2D (Fig. 5) and 3D (Fig. S3) confocal microscopy images showed a dense bacterial population in the growth control. Following treatment, the ISP-treated porcine skin showed reduced bacterial density, whereas RPCSa2-treated porcine skin showed bacterial density similar to the growth control. Consistent with the Gram-staining results, the greatest reduction in bacterial levels was observed with prophylaxis. In the human skin model, both the 2D (Fig. 6) and 3D (Fig. S4) confocal microscopy images showed a lower bacterial density in the growth control compared with the porcine skin model. Similar to the porcine skin, reduced bacterial levels were observed for the ISP-treated human skin but not for RPCSa2-treated skin, and the largest reduction in bacterial levels was observed with prophylaxis. After RPCSa2 treatment on the human skin, some of the living bacterial cells (green) seemed to be surrounded by a red signal. Since propidium iodide binds DNA, the red signal surrounding the cells is most likely caused by the binding of wheat germ agglutinin (WGA), indicating the presence of β-N-acetyl glucosamine (see insert Fig. 6E; Fig. S5). This was not observed on the porcine skin.

### The role of phage resistance and biofilm formation in bacterial survival during phage treatment

Although complete bacterial lysis was achieved under planktonic growth conditions at phage concentrations of only 10^2^ and 10^3^ pfu/mL (Fig. S1), *ex vivo* phage efficacy was limited, despite the use of 3-5 log higher phage concentrations. This discrepancy between *in vitro* and *ex vivo* models could be due to the development of phage resistance (23). To investigate the role of phage resistance in bacterial survival in burn wounds, bacteria were isolated from porcine and human skin 24 h after phage treatment, and a spot test was performed in triplicate. Phage susceptibility of these bacteria to phage ISP or RPCSa2 was compared with the susceptibility of bacteria isolated from skin after fusidic acid treatment and of a fresh *S. aureus* culture. Bacteria isolated from porcine and human skin after phage or fusidic treatment were still sensitive to both phages (Fig. S6 and S7). However, in some of the replicates, the phage-treated bacteria did not grow in the LB top layer of the spot test (Fig. S6B and S7A). A spot test with a spot of the bacterial suspensions obtained from the ISP- and RPCSa2-treated skin samples showed that these still contained active phages (Fig. S8). The presence of these active phages likely prevented bacterial growth in the LB top layer of the spot test, showing that the bacteria were still sensitive to phages despite their survival on the skin.

**Fig 7:**
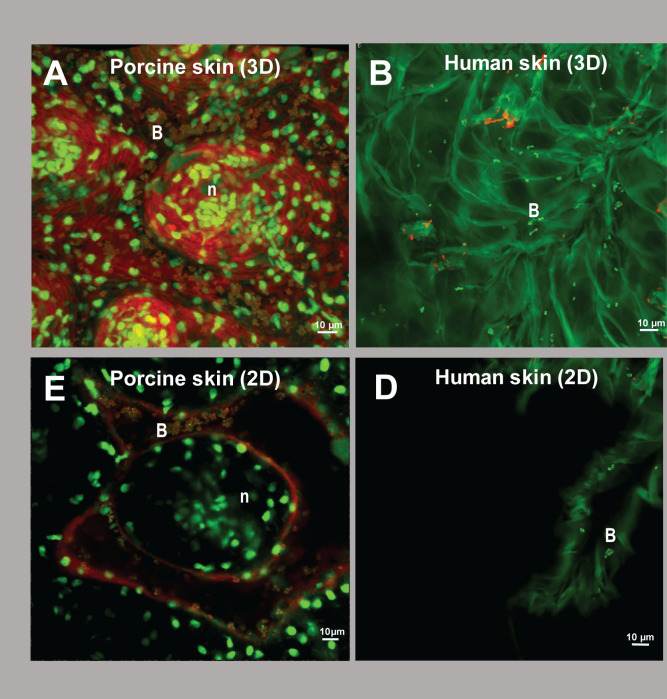
Fluorescent confocal microscopy images of burn wound infections 1 h after incubation with *S. aureus. S. aureus* was applied to (**A, C**) porcine skin or (**B, D**) human skin. After 1 h of incubation, planktonic cells were removed by washing, and dead bacterial and eukaryotic cells were stained with propidium iodide (red). Living bacteria (~1 µm) or eukaryotic nuclei (~10 µm) were stained with acridine orange (green). Additionally, WGA-594 (red) stained N-acetyl glucosamine, a sugar often produced in biofilms. All images were made using a 60× magnification objective. For clarity, two-dimensional cross-sections (**C and D**) are shown from the original three-dimensional images (**A and B**). B, bacteria; n, eukaryotic nuclei.

Biofilm formation can reduce phage sensitivity by shielding phage receptors on the bacterial surface (24). Since a higher reduction in bacterial growth was observed after prophylaxis compared with the phage treatment, we hypothesized that the 1-h incubation period before phage application might be enough to induce early biofilm formation, thereby reducing sensitivity to the phage treatment. To test this hypothesis, skin samples were imaged 1-h post-incubation with *S. aureus* using confocal microscopy (Fig. 7). The skins were washed to remove any planktonic bacteria before imaging. Both porcine skin and human skin showed bacteria residing in the crevices, but a higher density of bacteria was observed on the porcine skin compared with the human skin (Fig. 7A and C). The bacteria on the porcine skin were clearly clustered together and were surrounded by red signal, whereas the inside of the cells was stained green. The absence of a red signal within the cells indicated that the bacteria were alive, since propidium iodine was not able to penetrate the cell membrane. Therefore, the red signal surrounding the bacteria suggests binding of WGA, and thus, the presence of β-N-acetyl glucosamine after 1 h (Fig. 7C). For the human skin, no red signal surrounding the cells was observed, indicating an absence of WGA. Although most bacterial cells exhibited a clear green color, some were fully stained red, indicating cell death (Fig. 7B and D).

## DISCUSSION

The *in vivo* efficacy of phage therapy on *S. aureus* burn wound infections remains heavily understudied. To the best of our knowledge, there are currently no published *in vivo* animal studies on phage efficacy on *S. aureus* burn wound infection. In addition, only a limited number of clinical trials have been published thus far, which have shown the need for careful investigation of treatment regimens and read-out strategies (9, 10). In this study, two *ex vivo* burn wound infection models were used to investigate phage treatment for *S. aureus* burn wound infections. The human *ex vivo* burn wound model was used for this purpose for the first time. The bacterial load needed to cause an infection, and not merely colonization, is often debated. However, most studies suggest a threshold of 10^4^ to 10^5^ CFU/gram, which needs to be surpassed in order to speak of an infection (25). In this study, after inoculation with 10^5^ CFU, bacterial loads of the growth controls exponentially increased over time reaching 10^8^ to 10^9^ CFU in both models. After phage treatment, however, both models showed a significant reduction in bacterial load compared with the growth control, with bacterial loads below 10^4^ to 10^5^ CFU after 2 and 4 h. On human skin, a 2-3 log reduction of the bacterial load was observed, 24 h after a single phage treatment, resulting in bacterial loads of around 10^4^ to 10^5^ CFU. However, since these bacterial loads approached the threshold needed for infection and the assay duration was limited to 24 h, it remains difficult to conclude if the observed effects of the phage treatment will be clinically relevant.

Fusidic acid was outperformed by bacteriophage treatment, despite being added at a concentration of 30× its MIC. It should be noted that it might not reach this concentration locally, when spread out over the burn wound, thereby reducing its efficacy. In addition, fusidic acid is usually prescribed for 1 or 2 weeks to treat infections (National Health Service, 2021), whereas in this study, treatment efficacy was assessed after 24 h. Moreover, fusidic acid is a bacteriostatic antibiotic, which relies on the immune system to fully eliminate infections (6, 26). Although both *ex vivo* models contain most cell types present *in vivo*, they lack the influx of fresh immune cells via the blood. Therefore, these models could give an underestimation of the efficacy of fusidic acid. However, this underestimation may also apply to phage treatment, especially since phages have been shown to act synergistically with the (native) immune system to clear infections (27, 28). Although bacteria were still present after phage treatment, the reduction in bacterial load could still lead to full eradication with the contribution of the immune system (29). Similarly to fusidic acid, much higher phage concentrations were needed to reduce the bacterial load *ex vivo* compared with the *in vitro* assay. This discrepancy should be considered when using *in vitro* assays to predict phage and antibiotic efficacy *in vivo*.

An important limitation of phage therapy is the development of phage resistance (17). However, in this study, phage resistance did not significantly impact bacterial survival, as bacteria isolated from the skin after treatment showed equal susceptibility to phage treatment as the controls. However, since phage susceptibility was determined *in vitro*, we cannot rule out a contribution of transient phenotypic modifications, such as receptor modifications. These transient modifications often depend on environmental conditions, potentially leading to variations between *ex vivo* and *in vitro* assays and influencing phage sensitivity (30–32). In addition, this model only allows monitoring of phage efficacy for 24 h, whereas phage resistance may take days or even weeks to develop (33, 34). In the future, sequencing of both the active phages and surviving bacteria isolated from the skin should also be considered to gain further insights into phage-host dynamics and phage resistance in burn wounds (35).

The use of multiple successive treatments resulted in higher efficacy for phage RPCSa2 in the human model, but not for phage ISP or either phage in the porcine model. The limited additional effect of multiple treatments corresponds to the findings of Alves et al. (36), who also described the lack of benefit of an extra phage treatment compared with a single treatment using an *ex vivo* porcine model (36).

Prophylaxis resulted in a higher reduction of bacterial load compared with post-infection treatment, which was observed in both models even after 24 h. The increased efficacy of phage prophylaxis has been described previously in various pre-clinical *in vivo* studies (37–40). Onsea et al. (37) suggested that the advantage of prophylactic application of phages could be the prevention of biofilm formation, which is known to limit bacterial sensitivity to antibiotics and can shield bacterial phage receptors, thereby preventing phage infection (41). Since *S. aureus* is known to form biofilms on various surfaces, including the skin, we investigated the role of biofilm formation in the disparities in efficacy between pre- and post-infection phage treatments (41, 42). Confocal microscopy after 1 h of incubation showed bacterial attachment on the skin, persisting after washing, indicative of initial biofilm formation. Bacteria on the porcine skin were more abundant and clustered than on the human skin. In addition, WGA binding surrounding the living bacterial cells indicated the presence of N-acetyl glucosamine, which was absent on the human skin at this time point. The presence of N-acetyl glucosamine on the porcine skin may suggest the initiation of biofilm formation (41–43). Since biofilms are less sensitive to both antibiotics and phages (41–43), biofilm formation could explain the lower treatment efficacy for the porcine skin compared with the human skin, for which N-acetyl glucosamine could not be detected at this early time point. Moreover, the prevention of biofilm formation by prophylactic treatment could also explain the larger effect of phage prophylaxis on the porcine skin.

While porcine skin is very similar to human skin, there are still considerable differences (44). For example, the stratum corneum is thicker in humans than in pigs (45), and structural characteristics of the burned skin, such as skin permeability and lipid content, also differ (44). In this study, we also observed the inherent lack of nuclei in the human upper skin cells and the increased thickness of the porcine skin. The increased thickness resulted in deeper crevices in the porcine skin, where most bacteria resided. These bacteria might be less exposed to the forces of washing, which has been shown for bacteria that reside in fruit skin crevices (46). Therefore, protection of the bacteria in these crevices can reduce the effect of treatment with fusidic acid and phages. Another difference between the two models was the higher bacterial load observed in the growth control on porcine skin compared with human skin. This was evident in both the CFU data and the histology and confocal microscopy images, but the underlying mechanism for this observation is not understood.

Previous experiments with porcine *ex vivo* skin models have also shown a reduction in bacterial load after phage treatment of various bacterial species, including *P. aeruginosa, A. baumannii,* and *S. aureus* (5, 13). Although both models used in this study showed similar patterns of phage efficacy, our results show that the porcine skin model could lead to an underestimation of phage efficacy. However, human skin is harder to obtain in large quantities, making it less suitable than the porcine model for larger studies needed to optimize phage treatment with regard to phage strains, phage resistance, doses, and timing of treatment.

Altogether, this proof-of-principle study indicates that phage treatment can be effective in treating burn wound infections caused by *S. aureus*. The greatest impact of phage treatment is obtained when used as prophylaxis; however, in clinical practice, the causative agent of an infection is not known beforehand. Therefore, additional studies are needed to explore the prophylactic effect of phage cocktails targeting various epidemiologically relevant pathogens (37). The use of a phage cocktail not only broadens the host range but could also increase treatment efficacy compared with single phages (11). Other potential future studies include combining phage therapy with current burn wound infection treatment strategies, such as systemic antibiotic treatment or other topical antimicrobial agents (e.g., mupirocin or silver sulfadiazine) (5). In addition, alternative phage application strategies could be assessed, such as the use of encapsulated phages or phage-immobilized wound dressings to increase retention time and thereby phage efficacy (47, 48). The two *ex vivo* models described here are valuable tools for such future studies, which, together with this study, might pave the way for a better understanding of phage efficacy and the use of phage therapy for burn wound infections in clinical practice.

## MATERIALS AND METHODS

### Bacterial isolates and bacteriophages

#### Bacterial isolates

Throughout this study, methicillin-resistant *S. aureus* LUH14616 was used (sequence type 247), which is a clinical isolate from the nose of an *S. aureus* carrier (49, 50). Nasal carriage of *S. aureus* has been shown to be a risk factor for burn wound infections, with often a clonal relation between infectious and nasal strains (51). In addition, LUH14616 has previously been used to successfully infect thermally wounded human skin equivalents in multiple studies (49, 50). *S. aureus* strain R5 was used for phage propagation, since this strain is highly sensitive to phages and has historically been used for phage propagation for phage typing (21). Both bacterial strains were stored in glycerol at −80°C until use. Prior to experiments, bacteria were sub-cultured on Tryptic Soy Agar II plates with 5% sheep blood (TSA II) (BD, Franklin Lakes, USA) overnight at 37°C.

#### Bacteriophages

Bacteriophage RPCSa2 was previously isolated from a phage cocktail produced in Russia (NPO Microgen, Novgorod, Russia) and is closely related to polyvalent phage K. It has been shown to lyse a broad range of methicillin-susceptible and methicillin-resistant *S. aureus* strains (52). Bacteriophage ISP was kindly provided by Prof. Dr. Rob Lavigne (KU Leuven, Belgium) and was originally isolated in the Eliava Institute of Bacteriophage, Microbiology, and Virology in Georgia from an unknown source in the 1920s. Phage ISP has a broad host range, including MRSA strains and *S. aureus* burn wound isolates (53). In addition, this phage was included in a phage cocktail that has been used to empirically treat burn wound infections (10).

### Bacteriophage production

R5 was grown in 100 mL Tryptic soy broth (TSB), and 20 µL of either phage ISP or RPCSa2 was added when the bacteria reached the exponential growth phase (OD_600_ = 0.5 ± .2) and incubated overnight shaking at 37°C. The culture was centrifuged at 4,000 × *g* for 40 min at 4°C. Supernatant was recovered and filtered using a 0.22 µm Whatman puradisc filter (Merck KGaA, Darmstadt, Germany). The filtered suspension was further purified and concentrated using Zeba Spin Desalting columns with a 40 kD cutoff (ThermoFisher Scientific, Waltham, USA) and the PEG virus precipitation kit (Abcam, Cambridge, UK) according to the manufacturer’s protocols. The precipitated virus was re-suspended in SM buffer (100 mM NaCl, 8 mM MgSO_4_.7H_2_O, and 1 M Tris-CI, pH 7.5).

Phage titers were determined through a spot test as described previously (52). In short, a single colony of R5 was incubated in Luria-Bertani (LB) broth (Merck KGaA, Darmstadt, Germany) at 37°C and grown until the exponential phase. Next, 200 µL of bacteria was added to 3 mL 0.35% LB agar (containing 1M CaCl_2_ and 1M MgSO_4_) and poured onto a 1.4% LB agar plate. Ten-fold dilutions of each phage were prepared in phosphate-buffered saline (PBS). When the 0.35% LB agar solidified, 10 µL of each phage dilution was pipetted onto the plate. After overnight incubation at 37°C, plaque-forming units (pfu)/mL were determined.

### *In vitro* phage and antibiotic susceptibility determination

To determine the *in vitro* efficacy of phage ISP and phage RPCSa2, an absorbance (OD_600_) assay was performed (18). Bacteriophages were diluted 10-fold in SM buffer, with final concentrations ranging from 10^2^ to 10^8^ pfu/mL. A 0.5 McFarland suspension of *S. aureus* LUH1416 was prepared and diluted in LB broth (Merck KGaA, Darmstadt, Germany) to a final concentration of 10^6^ CFU/mL. Fifty microliters of the phage dilutions were added to 100 µL of the bacterial suspension and 50 µL PBS in a 96-well plate (Greiner, Kremsmüster, Austria) and incubated at 37°C. After 24 h, the absorbance (OD_600_) was measured using an Agilent BioTek Epoch 2 microplate spectrophotometer (ThermoFisher Scientific, Waltham, USA). Three independent replicate experiments were performed, and graphs were made with Graphpad Prism (v9).

Sensitivity of LUH1416 to fusidic acid (Sigma-Aldrich, Saint Louis, USA) was determined in the same manner with 50 µL of 2-fold dilutions of fusidic acid prepared in PBS (0.08 µg/mL to 20 µg/mL).

### Establishment of burn wounds on *ex vivo* skin samples

#### Human skin

Skin was processed as described by Dijksteel et al. (54). In short, human skin was obtained after elective surgery at the Red Cross Hospital (Beverwijk, the Netherlands) according to institutional guidelines and following “code of conduct for responsible use” drafted by Federa (Foundation Federation of Dutch Medical Scientific Societies). Human skin grafts with a thickness of 0.5 mm were prepared from this tissue, using a dermatome (Aesculap AG & Co. KG, Tuttlingen, Germany). Subsequently, the graft was cut into pieces of approximately 1 cm^2^. Skin pieces were stored at 4°C in RPMI (ThermoFisher Scientific, Waltham, USA). Burn wounds were applied with a copper device (2 × 10 mm) attached to an HQ soldering iron (Niehoff, Denekamp, the Netherlands), which was heated to 85°C and applied for 30 seconds to the stratum corneum without exerting pressure. The temperature of the copper device was measured by a digital thermometer (Farnell InOne, Utrecht, the Netherlands). The burned skin samples were placed dermis down on a stainless-steel grid and cultured at the air–liquid interface at 37°C with 5% CO_2_ in Dulbecco’s Modified Eagle’s Medium (ThermoFisher Scientific, Waltham, USA) with Ham’s F12 (3:1) (Invitrogen, Paisley, UK) (Fig. S9A).

#### Porcine skin

Porcine skin was collected from a slaughterhouse (Westfort, IJsselstein, the Netherlands). The skin was shaved, and excess fat tissue was removed, resulting in a skin graft with a thickness of roughly 3 mm. Subsequently, the graft was cut into pieces of approximately 1 cm^2^ using a scalpel. The skin was stored at 4°C in RPMI 1640 with L-Glutamine and HEPES (25 mM) (Capricorn Scientific, Ebsdorfergrund, Germany) with 1% penicillin-streptomycin (10.000 units/mL) (Lonza, Basel, Switzerland) for at least 2 days to remove residential skin flora. To prevent bacterial growth, the medium was refreshed every 4 days. Before applying the burn wound, the skin was submerged in 70% EtOH for 20 min, air-dried, and washed three times with PBS. Afterward, burn wounds were applied as described for the human skin, at a temperature of 100°C. One mL of DMEM with glucose (4.5 g/mL) (Capricorn Scientific, Ebsdorfergrund, Germany) was added to a six well plate, in which the skin samples were placed dermis down. The thickness of the skin samples allowed culturing at 37°C at the air–liquid interface without the additional use of a stainless-steel grid (Fig. S9B).

### *Ex vivo* phage and antibiotic susceptibility determination

#### Infection of burn wounds with *S. aureus* strain LUH14616

One colony of LUH14616 was grown in 5 mL of LB or TSB for human and porcine skin, respectively, until an OD_600_ of 0.5 (± 0.1) was reached. The bacterial cultures were stored overnight at 4°C and were placed back at 37°C the following day to restore the OD_600_ to 0.5 (± 0.1). The skin was prepared as described above. Although the exact number of CFU needed to cause an infection is subject to debate, bacterial loads between 10^4^ to 10^5^ CFU/g are suggested to cause infection by most studies (25). Therefore, a stock suspension of LUH14616 was prepared by diluting the bacterial culture (OD_600_ 0.5) in PBS, resulting in a concentration of 2 × 10^7^ CFU/mL. Subsequently, 5 µL of the bacterial stock suspension was added to each burn wound, resulting in 10^5^ CFU/burn wound. As a negative control, 5 µL of PBS was applied to the skin instead of bacteria. After application of LUH1416, skin samples were incubated at 37°C for 1 h, before treatment with phages or antibiotics.

#### Treatment

A 10-fold dilution series of phage RPCSa2 and ISP was made in SM buffer to achieve final concentrations of 3 × 10^8^, 3 × 10^7^, or 3 × 10^6^ pfu/skin. For each treatment, 10 µL of RPCSa2 or ISP or fusidic acid (10 µg/mL) was added to the skin at t = 0. To test the effect of multiple treatments, the treatment was repeated at t = 3 and t = 6 h. As a positive control for bacterial growth, 10 µL of SM buffer was added. The skin was then incubated at 37°C for 2, 4, or 24 h, after which the CFU was determined. For prophylaxis, phages were added to the burn wound and incubated for 1 h at 37°C before the application of LUH14616. After inoculation with LUH14616, the skin was incubated for 24 h at 37°C, after which the skin was processed, and the CFU was determined as described below. Experiments were performed in technical triplicates. Additionally, the single treatment t = 24 h has been performed as technical and independent replicates (*n* = 3).

#### Skin processing and CFU determination

Human tissue was transferred to polypropylene vials containing 1 mL of PBS and a 7 mm metal bead. In addition, 3 mM ferrous ammonium sulfate (FAS) (Sigma-Aldrich, Saint Louis, USA) was added for phage inactivation. This was the maximum concentration of FAS that could be added without interfering with bacterial growth. However, not all phages were inactivated at this concentration (data not shown). The tissue was homogenized using TissueLyser LT (Qiagen, Venlo, the Netherlands) set at 50 Hz for 5 min. Subsequently, the homogenate was diluted 10-fold, and 20 µL was added to an LB agar plate. After overnight incubation at 37°C, the CFU/mL was determined (54).

Porcine skin samples were transferred to 15 mL tubes containing 2 mL of PBS with 3 mM FAS and a ¼ inch ceramic sphere (MP Biomedicals, Burlingame, USA). Tissue homogenates were prepared using the FastPrep-24 tissue homogenizer (MP biomedicals, Burlingame, USA) at 6.5 m/s for 1.5 min. The supernatant was then diluted ten-fold, and 20 µL was plated on a TSA II plate. After overnight incubation at 37°C, the CFU/mL was determined.

Graphs were made with Graphpad Prism (v9). Conditions for which bacteria were completely absent were set to 1 to show them on a log scale. Data were log-transformed, and independent *t*-tests were performed to compare every condition with the growth control of the corresponding time point.

### Determination of bacteriophage resistance

Burn wound *S. aureus* infections were established as described above and treated with 3 × 10^8^ pfu/mL phage ISP or RPCSa2. Fusidic acid-treated samples (500 µg/mL) were included as a negative control for phage resistance. After 24 h, the skin was processed for CFU counting. In addition, 100 µL of the dilutions were plated on TSA II plates and incubated overnight at 37°C. When bacterial growth was observed, three colonies per plate were pooled and used to perform a spot test. As an additional control for phage susceptibility, a fresh LUH14616 culture was included in the spot test. If the isolated colonies did not grow to OD_600_ 0.5, bacteria were suspended directly from the plate to achieve OD_600_ 0.5 (±0.1). In addition, the bacterial suspensions were examined for the presence of active phages. For this purpose, 10 µL of the bacterial suspensions were pipetted on top of agar containing the fresh LUH14616 culture. For all conditions, plaque-forming units were determined after an overnight incubation at 37°C.

### Visualization of treatment efficacy

#### Gram staining

Skin samples were fixed using 4% paraformaldehyde and stored in PBS at 4°C. Vertical slices of the formalin-fixed paraffin-embedded (FFPE) tissue were made on which Gram-staining was performed (55). For each condition, the number of bacteria present per frame was determined, with a total of 20 frames per condition at 400× magnification. The number of bacteria within one frame was scored in four categories: no bacteria, 1 to 10, 10 to 100, and more than 100 bacteria. The percentage of frames belonging to each category was calculated (Table 1). A representative frame for each condition was imaged using an Axioskop microscope (Zeiss, Oberkochen, Germany) at 400× and 1000× magnification.

#### Confocal microscopy

To visualize the effect of phage treatment, the skin was stained with 200 µL propidium iodide (25 µg/mL) (ThermoFisher Scientific, Waltham, USA) to visualize dead cells, and 200 µL of a 0.01% solution of acridine orange (ThermoFisher Scientific, Waltham, USA) to image live cells (19, 20). In addition, 200 µL WGA-Alexa fluor-594 (74 µg/mL) (Invitrogen, Paisley, UK) was added to visualize β-N-acetylglucosamine, which accumulates in biofilms (41, 56). For each condition, two skin samples were prepared and imaged using a custom-built, upright Nikon A1R + confocal microscope with a 20× magnifying objective. Three images were taken per sample, one representative is shown.

To investigate bacterial aggregation and attachment to the skin after 1 h of incubation, 1 × 10^5^ CFU of *S. aureus* LUH14616 was added to the skin and incubated for 1 h at 37°C. The skin was then washed three times with PBS to remove any planktonic bacteria, stained, and imaged with a 20× and 60× magnifying objective as described above.

## SUPPLEMENTAL MATERIAL

Supplemental material aac.00650-24-s0001.docx Figures S1 to S9.

ASM does not own the copyrights to Supplemental Material that may be linked to, or accessed through, an article. The authors have granted ASM a non-exclusive, world-wide license to publish the Supplemental Material files. Please contact the corresponding author directly for reuse.
