# Supplementary material for: Bacteriophage therapy reduces Staphylococcus aureus in a porcine and human ex vivo burn wound infection model
Source: Antimicrob Agents Chemother. 2024 Aug 13;68(9):e00650-24. doi: 10.1128/aac.00650-24 (PMC11373223; doi:10.1128/aac.00650-24)
Supplement: Supplemental material — Figures S1 to S9. [file aac.00650-24-s0001.docx]

**Supplemental figures**

**Supplemental Figure S1: *In vitro* susceptibility of *S. aureus* LUH14616 to phages and fusidic acid.** The optical density (OD_600_) was measured in three independent experiments after twenty-four hours of incubation of *S. aureus* with various concentrations of either A) phages (pfu/mL) or B) fusidic acid (µg/mL). No bacterial growth was measured after the addition of phage ISP and phage RPCSa2 at 10^2^ pfu/mL and 10^3^ pfu/mL, respectively. Fusidic acid completely abolished bacterial growth at concentrations higher than 0.16 µg/mL, indicating a minimal inhibitory concentration of 0.31 µg/mL.


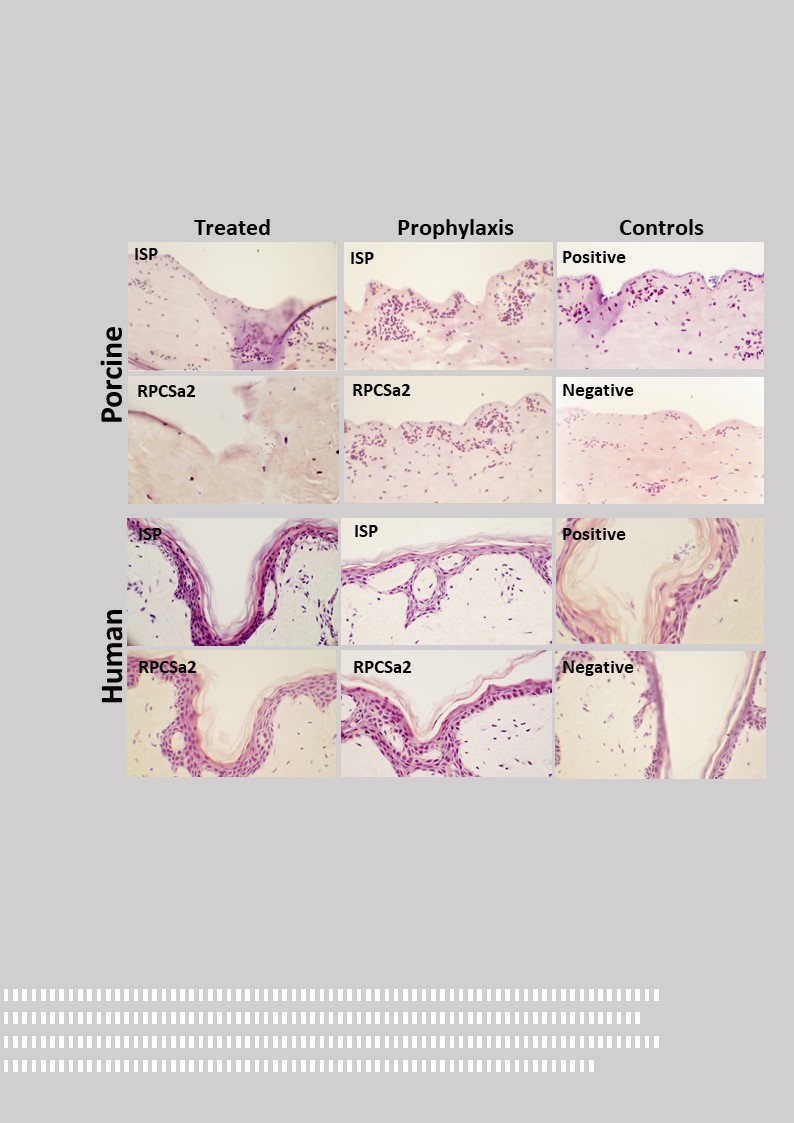


**Supplemental Figure S2: Gram-staining of porcine and human skin after phage treatment or prophylaxis (400x magnification).** Images of Gram-stained skin tissue twenty-four hours after phage treatment or prophylaxis with 10^8^ pfu/mL of phage ISP or RPCSa2. A positive control (untreated) and negative control (no bacteria added) were included for comparison.


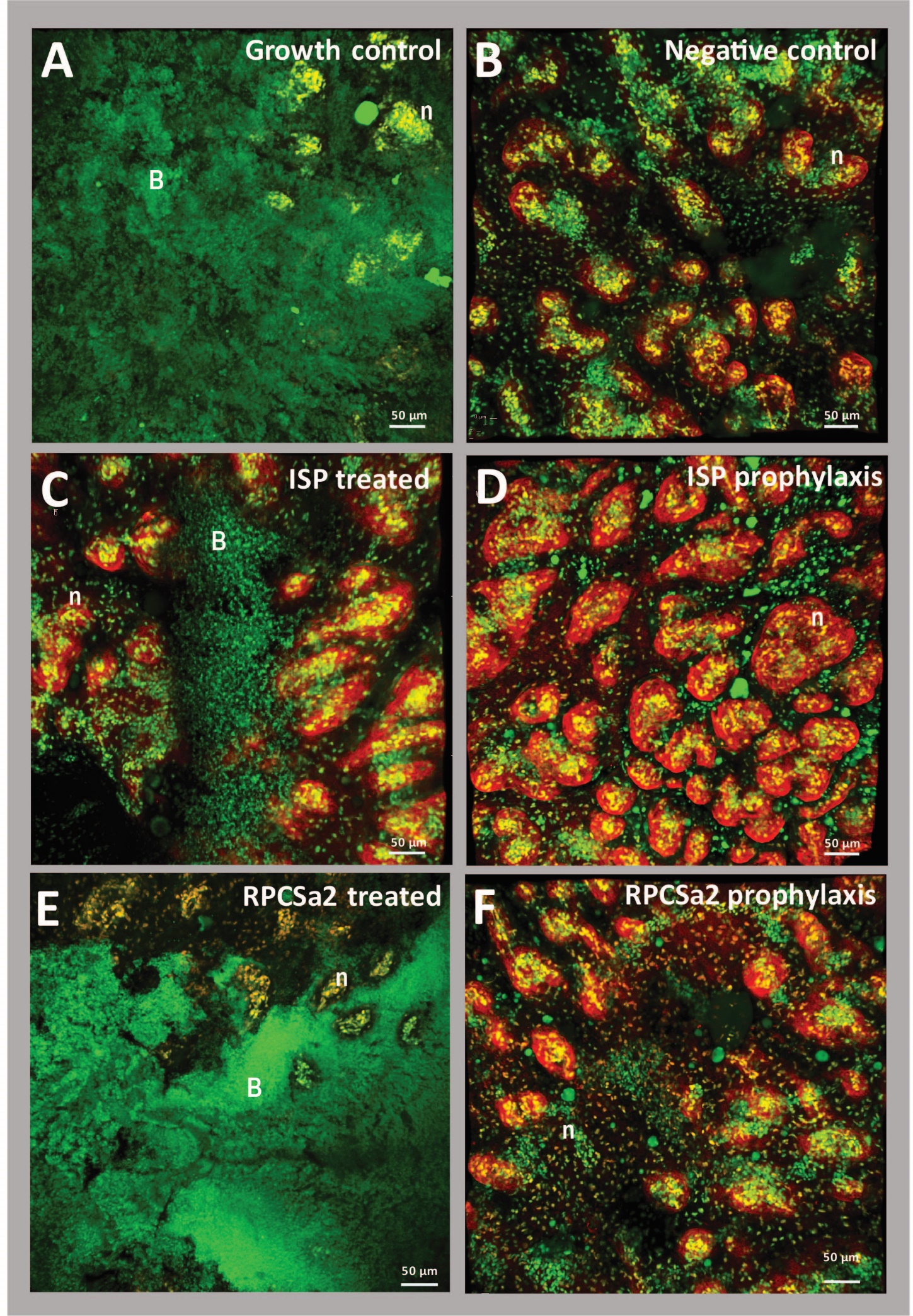


**Figure S3: Three-dimensional confocal microscopy images of porcine skin twenty-four hours after phage treatment or prophylaxis.** Images were made of porcine skin tissue twenty-four hours post treatment. Skin was incubated with *S. aureus* and was A) left untreated as a growth control, C) treated or D) prophylactically treated with 10^8^ pfu/mL ISP, E) treated or F) prophylactically treated with 10^8^ pfu/mL RPCSa2. Skin without bacteria was included as B) negative control. Dead bacterial and eukaryotic cells were stained with propidium iodide(red). Living bacteria (~1 µm) or eukaryotic nuclei (~10µm) were stained with acridine orange (green). Additionally, WGA-594 (red) stained N-acetyl glucosamine, a sugar often produced in biofilms. All images were made at twenty times magnification. B, bacteria; n, eukaryotic nuclei.


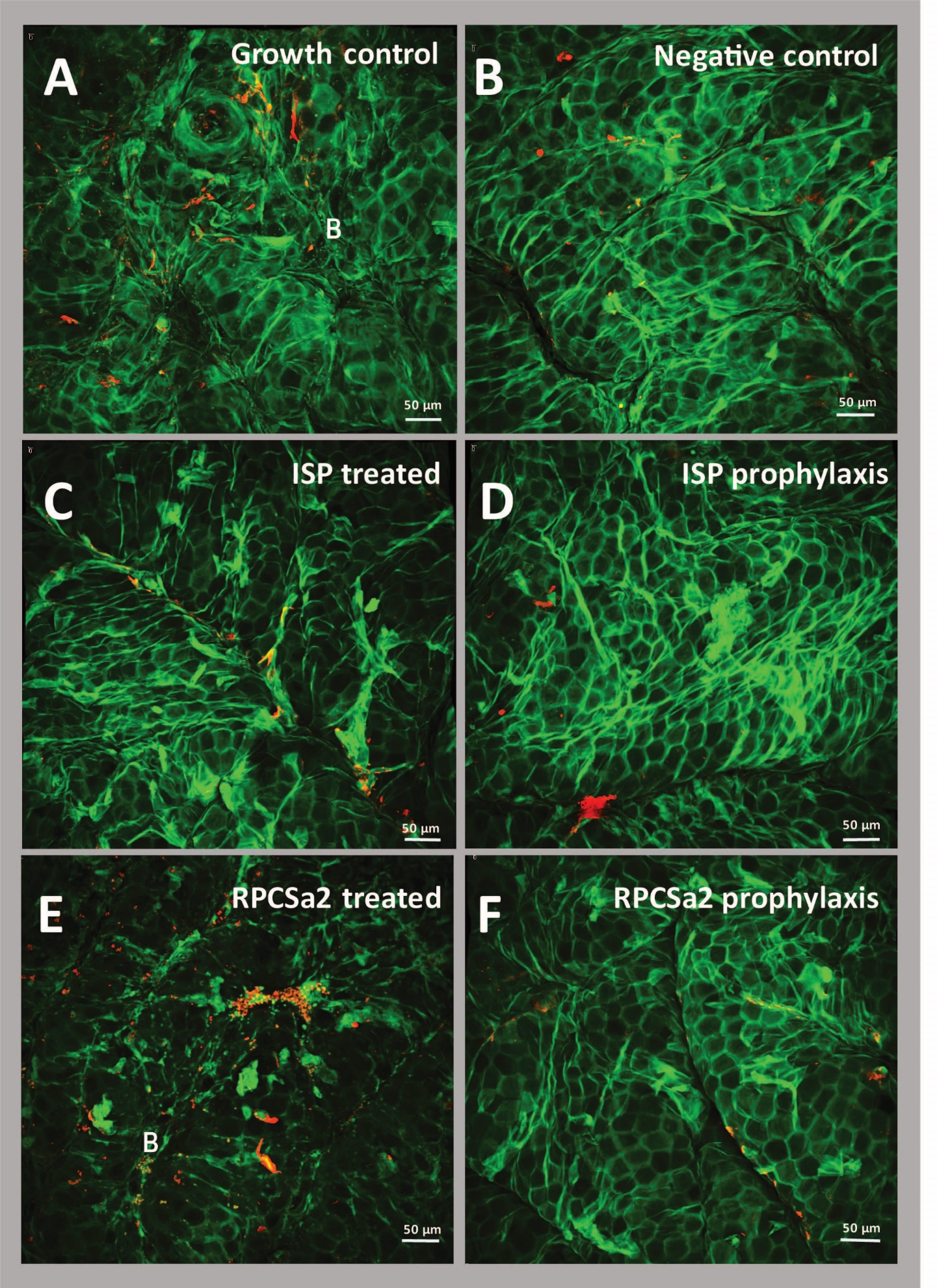


**Figure S4: Three-dimensional confocal microscopy images of human skin twenty-four hours after phage treatment or prophylaxis.** Images were made of human skin tissue twenty-four hours post treatment. Skin was either incubated with *S. aureus* and was A) left untreated as growth control, C) treated or D) prophylactically treated with 10^8^ pfu/mL ISP, E) treated or F) prophylactically treated with 10^8^ pfu/mL RPCSa2. Skin without bacteria was included as B) negative control. Dead bacterial and eukaryotic cells were stained with propidium iodide (red). Living bacteria (~1 µm) or eukaryotic nuclei (~10µm) were stained with acridine orange (green). Additionally, WGA-594 (red) stained N-acetyl glucosamine, a sugar often produced in biofilms. All images were made at twenty times magnification. B, bacteria.


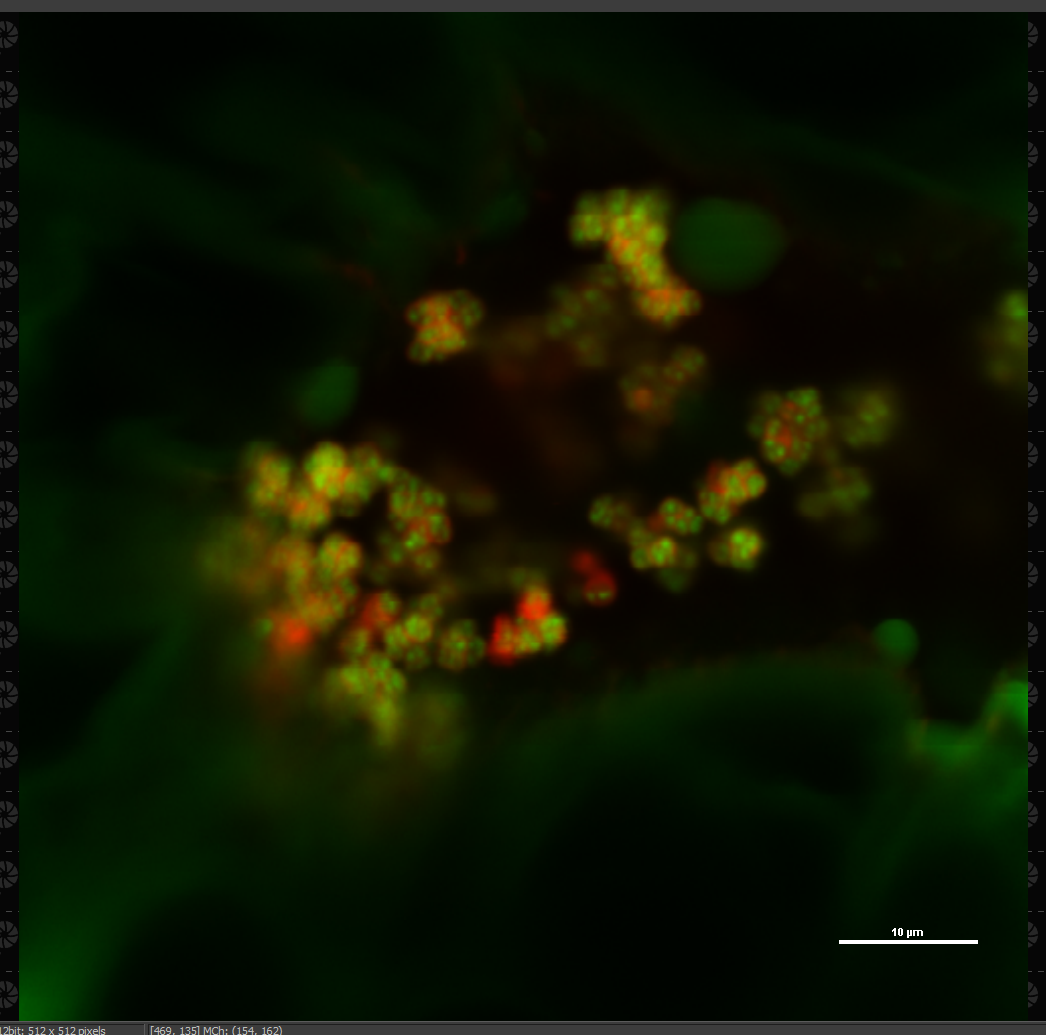


**10 µm**

**Figure S5: High resolution two-dimensional confocal microscopy image of human skin twenty-four hours after RPCSa2 treatment.** Skin was either incubated with *S. aureus* and was treated with 10^8^ pfu/mL RPCSa2. Dead bacterial and eukaryotic cells were stained with propidium iodide (red). Living bacteria (~1 µm) or eukaryotic nuclei (~10µm) were stained with acridine orange (green). Additionally, WGA-594 (red) stained N-acetyl glucosamine, a sugar often produced in biofilms.


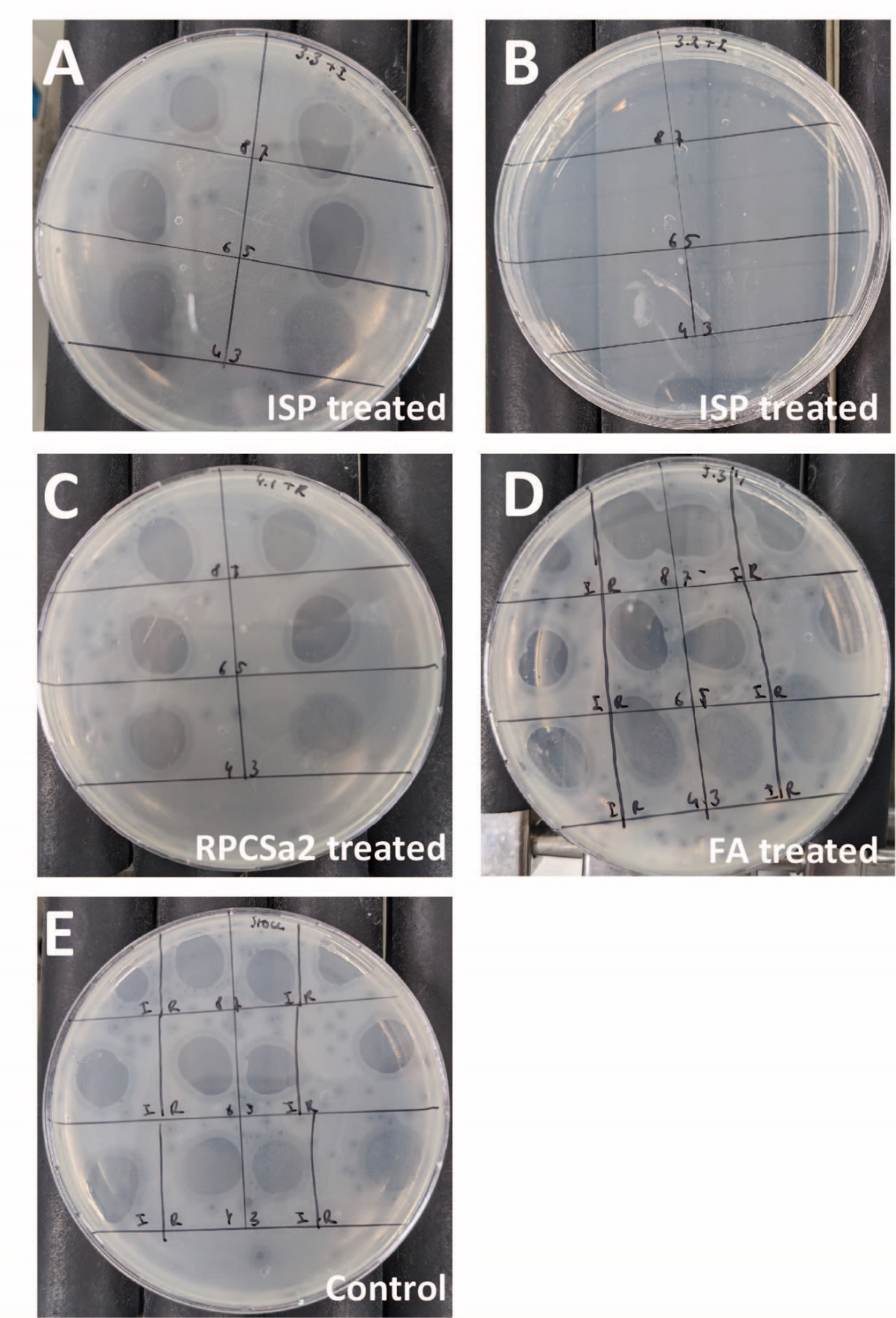


**Supplemental figure S6: Phage susceptibility of bacteria isolated from the porcine skin after phage treatment.** Bacteria were isolated from burn wounds twenty-four hours after treatment with A, B) 10^8^ pfu/mL ISP, C) 10^8^ pfu/mL RPCSa2, or D) 500 µg/mL fusidic acid (FA). As a control E) *S. aureus* colonies grown from a fresh culture were included. Phage susceptibility to ISP and RPCSa2 (10^8^ – 10^3^ pfu/mL) was determined using a spot test. All conditions were tested in triplicate and representative plates are shown. All three replicates of RPCSa2 treated bacteria showed similar phage susceptibility as the fresh culture control. B) For two of three replicates, ISP treated bacteria did not grow in the culture medium, disrupting the spot test. The other replicate showed similar phage susceptibility as the controls.


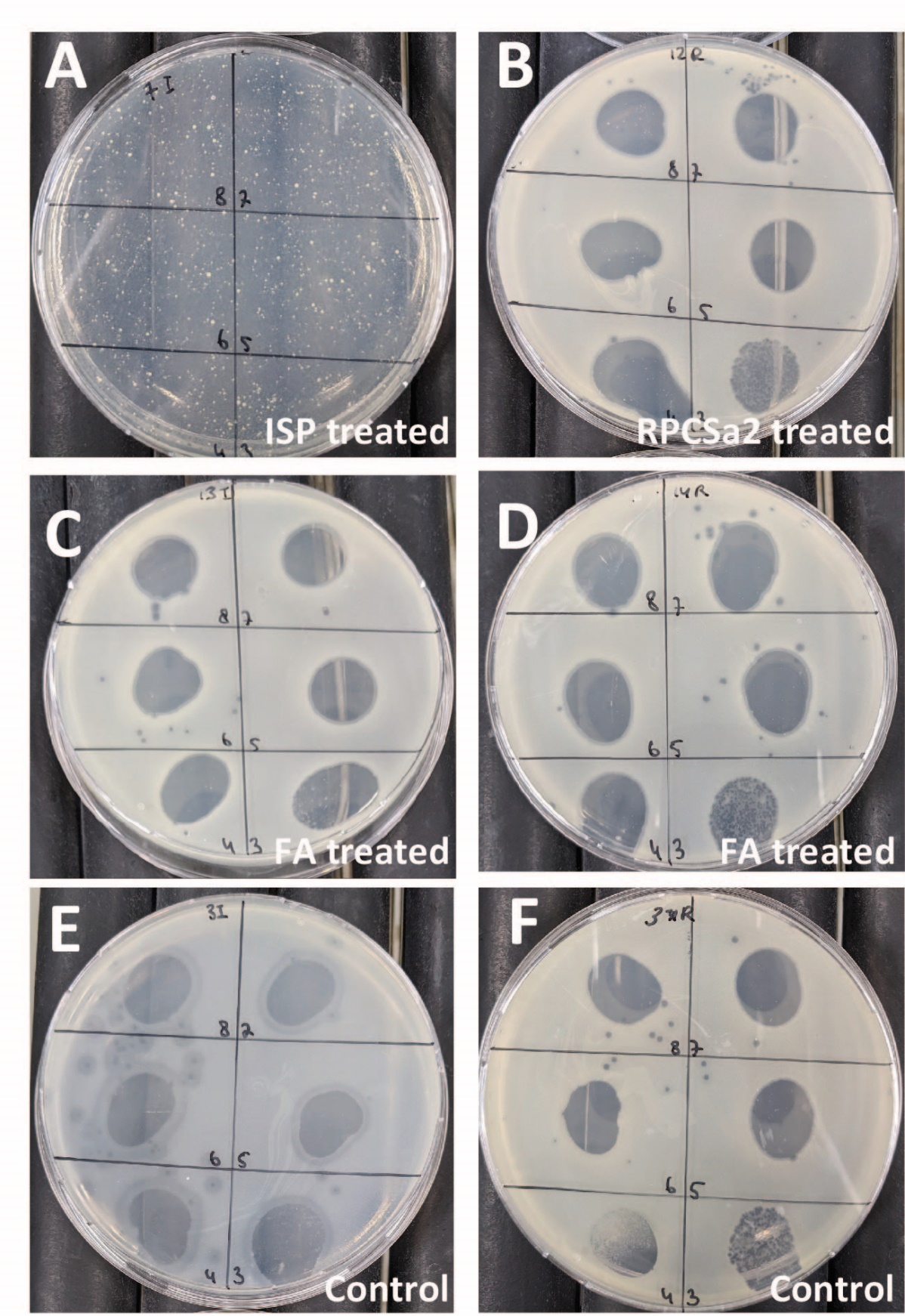


**Supplemental figure S7: Phage susceptibility of bacteria isolated from the human skin after phage treatment.** Bacteria were isolated from burn wounds twenty-four hours after treatment with A) 10^8^ pfu/mL ISP or B) 10^8^ pfu/mL RPCSa2. Phage susceptibility to ISP and RPCSa2 (10^8^  to 10^3^ pfu/mL) was determined using a spot test. In addition, bacteria isolated from burn wounds twenty-four hours after treatment with 500 µg/mL fusidic acid (FA) were included and susceptibility to C) ISP or D) RPCSa2 was determined. As a control, *S. aureus* colonies grown from a fresh culture were included and susceptibility to E) ISP and F) RPCSa2 was determined. One of the two replicates of bacteria isolated from the skin treated with RPCSa2 showed similar phage susceptibility as the *S. aureus* control plate. The other replicate and bacteria isolated from the skin treated with ISP did not grow in bacterial culture medium, disrupting the spot test (representative plate shown in A).


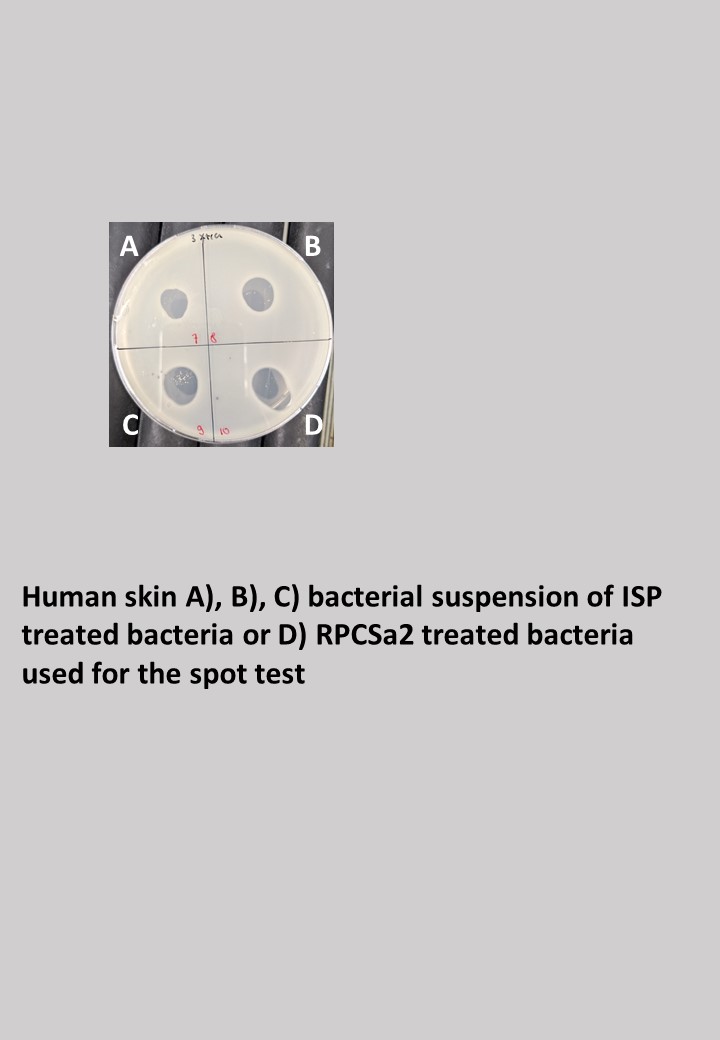


**Supplemental figure S8: Spot test with the bacterial suspension isolated from human skin.** A top layer containing *S. aureus* from a fresh culture with a droplet of A), B), C) the bacterial suspension from the burn wound treated with ISP or D) RPCSa2.

**
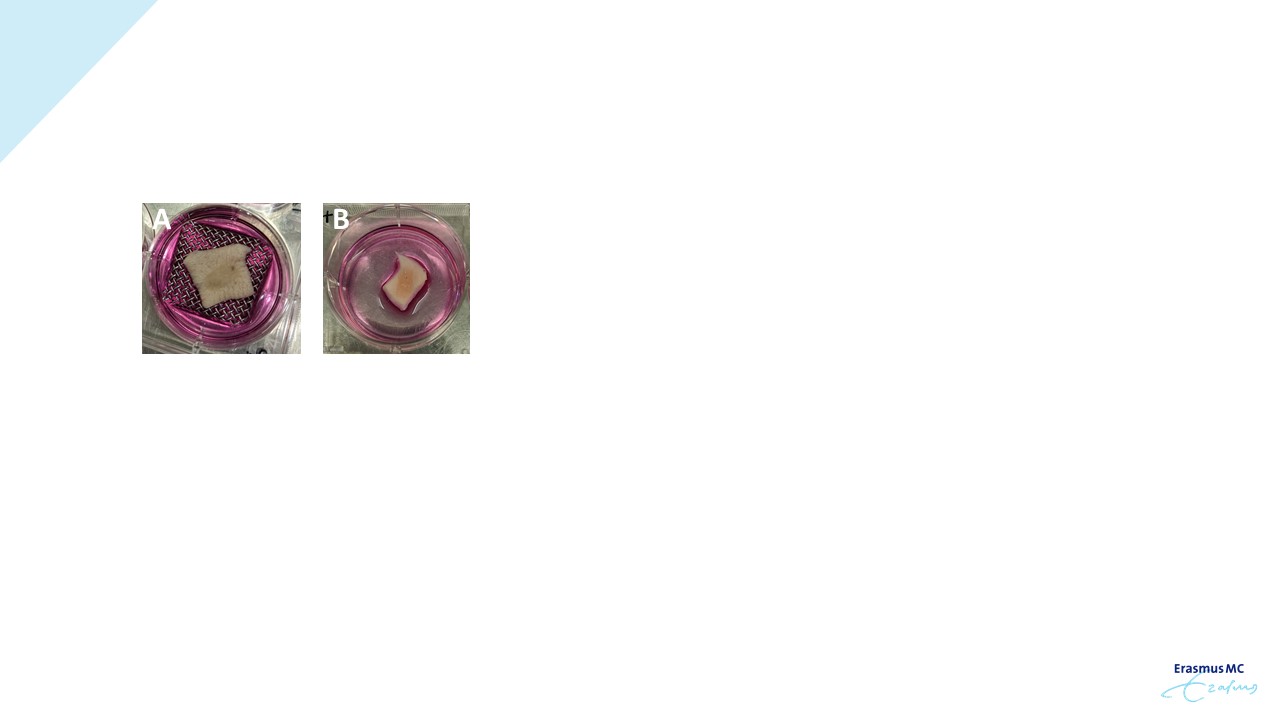
Supplemental figure S9**: **Image of human and porcine skin at the air-liquid interphase.**

A) human skin in a six-wells plate at air-liquid interphase using a stainless-steel grid, B) porcine skin in a six-wells plate at the air-liquid interphase. Burn wounds were applied to the center of the skin, resulting in a discoloring of the skin in the burned area.
